# NUSAP1 Promotes Gastric Cancer Tumorigenesis and Progression by Stabilizing the YAP1 Protein

**DOI:** 10.3389/fonc.2020.591698

**Published:** 2021-01-07

**Authors:** Hui Guo, Jianping Zou, Ling Zhou, Min Zhong, Yan He, Shanshan Huang, Jun Chen, Junhe Li, Jianping Xiong, Ziling Fang, Xiaojun Xiang

**Affiliations:** Department of Oncology, The First Affiliated Hospital of Nanchang University, Nanchang, China

**Keywords:** gastric cancer, NUSAP1, YAP1, protein stability, tumorigenesis and progression

## Abstract

The Yes-associated protein (YAP1) is a main effector of the canonical Hippo pathway, which contributes greatly to tumor initiation, progression, and metastasis in multiple cancers, including gastric cancer (GC). Due to limited knowledge of YAP1 upregulation in cancer, it is a great challenge of therapeutic targets toward the Hippo–YAP1 pathway. Here, we identify nucleolar spindle-associated protein 1 (NUSAP1) as a novel binding partner of YAP1. The upregulation of NUSAP1 is associated with unfavorable clinical outcomes in GC patients, and NUSAP1 depletion impairs its oncogenic properties *in vitro* and in a xenograft model. Mechanistically, we discovered that NUSAP1 functions as a positive regulator of YAP1 protein stability, thereby inducing the transcription of Hippo pathway downstream target genes, such as CTGF and CYR61. More interestingly, we find that the cancer-promoting effects of NUSAP1 on GC cell growth, migration, and invasion are mainly mediated by YAP1. Furthermore, aberrant expression of NUSAP1 and YAP1 is highly correlated in GC cell lines and tissues. We herein clarify the role of the oncogenic NUSAP1–YAP1 axis in GC tumorigenesis and progression and, therefore, provide novel therapeutic targets for GC treatment.

## Introduction

Gastric cancer (GC) is one of the most lethal malignancies worldwide and has a particularly high mortality rate in East Asian countries, especially in China and Japan (1). Despite great progress in surgical and comprehensive therapies, improvements in the clinical outcomes of patients with GC remain limited (2). Therefore, it is of utmost importance to explore the molecular mechanism underlying GC tumorigenesis and progression and to identify new therapeutic targets for patients with GC.

Numerous studies point to the pivotal role of the Hippo pathway in tissue growth and organ size by a delicate balance between cell proliferation and cell death (3). The core Hippo pathway consists of a kinase cascade: the upstream kinase MST1/2 phosphorylates and activates the downstream kinase LATS1/2, leading to phosphorylation and inactivation of the transcriptional coactivator Yes-associated protein (YAP1) (4–6). Then, the phosphorylated YAP1 translocates to the nucleus and associates with transcription factors, such as the TEA domain (TEAD) family, RUNX, and SMADs (7–9), consequently favoring an accelerated rate of cell growth, invasiveness, and survival. Upregulation of YAP1 expression and its nuclear translocation are frequently detected in several human cancers, including breast cancer, hepatocellular carcinoma, and GC (10–12). Although many studies have outlined that YAP1 overexpression or activation contributes greatly to GC tumorigenesis, the pathological mechanisms underlying YAP1 upregulation are still poorly understood.

Nucleolar spindle-associated protein 1 (NUSAP1) is a microtubule-associated protein that plays a critical role in various biological functions, including spindle assembly, chromosome segregation, cytokinesis, microtubule crosslinking, bundling, and attachment to chromosomes (13–15). Studies show that NUSAP1 is involved in human malignancies, including pancreatic adenocarcinoma, glioblastoma, hepatocellular carcinoma, prostate cancer, etc (16–20). High expression of NUSAP1 is positively correlated with poor prognosis in lung cancer and cervical carcinoma (21, 22). Although NUSAP1 is characterized as an oncogenic driver in several cancers, the underlying mechanisms remain elusive. Zhang et al. report that NUSAP1 depletion suppresses cell proliferation, migration, and invasion by regulating CDK1 and DLGAP5 expression in invasive breast cancer cells (23). Furthermore, the signaling pathways responsible for NUSAP1 functions include PI3K/Akt, Wnt/β-catenin, and Hedgehog (22, 24, 25), which are highly relevant to tumorigenesis. NUSAP1 is also identified to be upregulated in GC (26). Thus far, the biological functions of NUSAP1 and the underlying mechanisms in GC have not been well elucidated.

Here, we find that NUSAP1 plays an oncogenic role in gastric oncogenesis through interaction with YAP1 and promotes its nuclear translocation. Moreover, NUSAP1 is positively correlated with poor clinical outcomes and YAP1 protein expression in GC. Our findings not only establish a previously undocumented NUSAP1–YAP1 axis in driving GC carcinogenesis, but also provide therapeutic targets in personalized medicine ear for GC patients.

## Materials and Methods

### Ethics Statement and Clinical Tissues

Our study was approved by the Independent Ethical Committee of the First Affiliated Hospital of Nanchang University and complies with the Declaration of Helsinki. Written informed consent was received from all patients. Eight primary GC tissues and their corresponding normal gastric tissues were obtained from the Department of Surgery at the First Affiliated Hospital of Nanchang University. Upon resection, the fresh tissue samples were immediately frozen in liquid nitrogen and stored at -80°C. The clinicopathological characteristics of GC patients are summarized in **Supplementary Table S1**. A total of 161 paraffin-embedded GC samples were included in this study. All GC patients were treatment-naïve before surgery. The clinicopathological features of the GC patients were confirmed by two experienced pathologists at the First Affiliated Hospital of Nanchang University between 2012 and 2016. The clinical and pathological grade was conducted according to the eighth edition of the classification system of the American Joint Committee on Cancer (AJCC).

### Cell Lines and Cell Culture

The human GC cell lines AGS, BGC823, MGC803, HGC-27, SGC7901, and MKN45 and the immortalized gastric epithelial cell line GES-1 were purchased from the Shanghai Institute of Cell Biology, China Academy of Sciences. Cells were cultured in Dulbecco’s modified Eagle’s medium (DMEM, HyClone, Logan, UT, USA) with 10%–15% fetal bovine serum (FBS; HyClone, USA) at 37°C in an atmosphere containing 5% CO_2_. Cells were harvested at the indicated times posttransfection for future experiments.

### Vectors, Lentiviral Infection, and Transfection

Flag-NUSAP1 plasmid was generated by subcloning the PCR-amplified human NUSAP1 coding sequence into the 2×pcDNA3.1 vector at the NheI and HindIII sites. NUSAP1-targeting shRNAs and YAP1-targeting siRNA oligonucleotides were designed and synthesized by GenePharma. The sequences of shRNAs and siRNAs used in our study are provided in **Supplementary Table S2**. Cells were seeded on the plate the day before transfection and transfected with the indicated plasmids, shRNAs, and siRNAs using TurboFect transfection reagent (Thermo Scientific, R0532, USA). Lentivirus production and infection were conducted as described previously (27). Stable GC cell lines transfected with scramble shRNA or NUSAP1 shRNAs were selected for 2 weeks and incubated with 2 μg/mL puromycin upon infection.

### Co-Immunoprecipitation and Mass Spectrometry Analysis

The co-immunoprecipitation (Co-IP) assay was conducted utilizing indicated antibodies according to the figure legends as previously described (27). Briefly, 1000 μg of total protein was incubated with anti-Flag beads (#M185-11 MBL, Tokyo, Japan) or anti-HA beads (#ab18181, Abcam, Cambridge) at 4°C for 4 h. The beads were washed with lysis buffer at least three times. Bound proteins were detected by Western blotting analysis with indicated antibodies described in the figure legends.

Flag-YAP1 was transfected into BGC823 cells following the manufacturer’s instructions. Upon transfection for 36 h, cells stably expressing Flag-YAP1 or pcDNA were established by antibiotic selection. Then, 6×10^8^ Flag-YAP1 or control vector group cells were harvested and incubated with anti-Flag beads overnight at 4°C. The cell samples were subjected to SDS-PAGE gel, and the gel was eluted with Flag peptides with cold PBS. The bands in the YAP1 overexpression group were excised for in-gel trypsin digestion, peptide extraction, and mass spectrometry analysis.

### Western Blotting Analysis

Western blotting analysis was conducted using NUSAP1 (1:1000; #12024-1-AP; Proteintech, Rosemont, IL, USA), YAP1 (1:1500; #14074; Cell Signaling Technology, Danvers, USA), LATS1 (1:1500; #3477; Cell Signaling Technology, Danvers, USA), LATS2 (1:2000; #5888; Cell Signaling Technology, Danvers, USA), CTGF (1:2000; #86641; Cell Signaling Technology, Danvers, USA), CYR61 (1:2000; #14479; Cell Signaling Technology, Danvers, USA), anti-HA (1:2500; #ab9110; Abcam, Cambridge, USA), anti-Flag (1:2500; #A2220; Sigma-Aldrich, USA), β-actin (1:3000; #AF7018; Affinity, Jiangsu, China) and GAPDH (1:3000; #A2220; Affinity, Jiangsu, China). Human GC samples originally obtained from the First Affiliated Hospital of Nanchang University, along with the xenograft tumors, were ground and lysed in lysis buffer before Western blotting analysis. The Western blotting experiments were performed as previously described (28). GAPDH or β-Actin was used as an internal control, and all the protein bands were analyzed using ImageJ software.

### Immunohistochemistry

Immunohistochemistry was performed in 161 clinical GC samples as reported previously (29). The degree of immunostaining was assessed and scored by two pathologists in a blinded manner. The protein expression of NUSAP1 in GC specimens by determining the SI (the product of the staining intensity score and the proportion of positive cells) with possible scores of 0, 1, 2, 3, 4, 6, 8, 9, and 12. Samples with a score index ≥6 were considered to have high NUSAP1 expression, and those with a score index <6 were considered to have low NUSAP1 expression. The immunohistochemical staining and quantification of YAP1 protein were performed as previously described (30).

### RNA Extraction and Quantitative Real-Time PCR

Total RNA was extracted from cells or xenograft tissues using TRIzol (Invitrogen, Carlsbad, CA, USA) according to the manufacturer’s protocol as previously described (31). Total RNA (0.5 or 1.0 μg) was used as a template for reverse transcription using poly-(T)20 primers and M-MLV reverse transcriptase (Promega, Madison, USA). Quantitative RT-PCR (qRT-PCR) was performed using SYBR Green Mix following the manufacturer’s protocol (BioRad, Hercules, USA). The 2^–ΔΔCt^ method was utilized to assess the relative mRNA expression of genes among groups. The primer sequences used are listed in **Supplementary Table S3**.

### Cell Counting Kit-8 (CCK-8) Assay

To assess cell growth ability, the CCK-8 assay (Beyotime, China) was utilized following the manufacturer’s instructions as previously described (32). After transfection for 24 h, cells were inoculated in 96-well plates. Cell viability was measured by adding 10 μL CCK-8 reagent and incubated for 1 h; then, the optical density was measured at the absorbance of samples at 450 nm in a microplate reader (SpectraMax M5e, USA) for 5 days. The data derived from triplicate samples are shown as the mean ± standard error of the mean (SEM).

### Colony Formation Assay

The colony formation assay was performed as previously described (33). The indicated transfected HGC-27 and BGC823 cells were trypsinized and plated at equal numbers of cells into 6-well plates. Media were changed every 3–4 days until the colonies were visible. Colonies were fixed with 4% paraformaldehyde (PFA) and stained with 10% crystal violet at room temperature for 30 min. ImageJ software (Rawak Software, Stuttgart, Germany) was used for quantification of the colonies. These experiments were conducted more than three times.

### Wound-Healing Assay

The indicated HGC-27 and BGC823 cells were plated into 6-well plates at a concentration of 4.6 × 10^5^ per well and serum-starved for 24 h until completely confluent on the second day. A sterile 200 μL pipette tip was used to draw straight lines to form a wound. The cells were carefully washed with phosphate-buffered saline (PBS) and cultured in serum-free medium. Images were taken at 0, 24, or 36 h after scratching to evaluate wound closure. Each experiment was carried out for at least three times.

### Transwell Assay

The transwell chambers were prepared with Matrigel gel, and the indicated HGC-27 or BGC823 cells were plated into the upper chambers as described previously (30). After 72 h of incubation, cells passing through the chamber membranes were fixed with 4% PFA and stained with 10% crystal violet. These experiments were conducted using three biological replicates.

### Immunofluorescence Staining

The indicated BGC823 cells were fixed in 4% PFA for 15 min and then treated with 0.5% Triton X-100 for 10 min. Nonspecific bindings were blocked with 5% bovine serum albumin (BSA) for 30 min. The cells were incubated with the anti-Flag or anti-GFP antibodies at 4°C overnight and incubated with the corresponding secondary antibody for 20 min. The 4’-6-diamidino-2-phenylindole (DAPI) was used to stain the nuclei for 30 min. The cellular localization of NUSAP1 or YAP1 was detected using a confocal microscope (Nikon, ECLIPSE Ti2).

### Animal Models

Six- to seven-week-old female nude mice were purchased from the SLACCAS Experiment Animal Company (Shanghai, China), and 5.6×10^6^ indicated BGC823 cells were subcutaneously inoculated into the left axilla of each mouse. Tumor growth was monitored every 3 days with electronic digital calipers (Thermo Scientific) in two dimensions. Tumor volume was measured with the formula: tumor volume (mm^3^) = (length × width^2^)/2. After 28 days, the mice were sacrificed by euthanasia, and xenograft tumors were harvested and weighed. Total RNA was extracted from tumors *via* homogenization in TRIzol buffer and then subjected to Western blotting and qRT-PCR analysis. Our study was approved by the Ethics Committee of the First Affiliated Hospital of Nanchang University for animal research.

### Statistical Analysis

The SPSS 20.0 software (Chicago, IL, USA) was utilized to perform the statistical analysis. Student’s two-tailed *t* test or the chi-square test was used to determine the mean difference among groups. Survival curves were plotted using the Kaplan–Meier method and compared by the log-rank test. *P*<0.05 was considered statistically significant. The term ‘n.s’ indicates that no significant difference was found. All the data are presented as the mean ± SEM.

## Results

### NUSAP1 Interacts With YAP1

To explore the interactive components of YAP1, we conducted a pull-down and mass spectrometry analysis of YAP1-interacting proteins from BGC823 GC cells. The mass spectrometry results not only revealed several previously described YAP1-regulatory proteins, such as LATS1 and IRF3, as YAP1 binding proteins, but also identified NUSAP1 as a potential YAP1-binding protein (**Supplementary Figure S1**).

**Figure 1:**
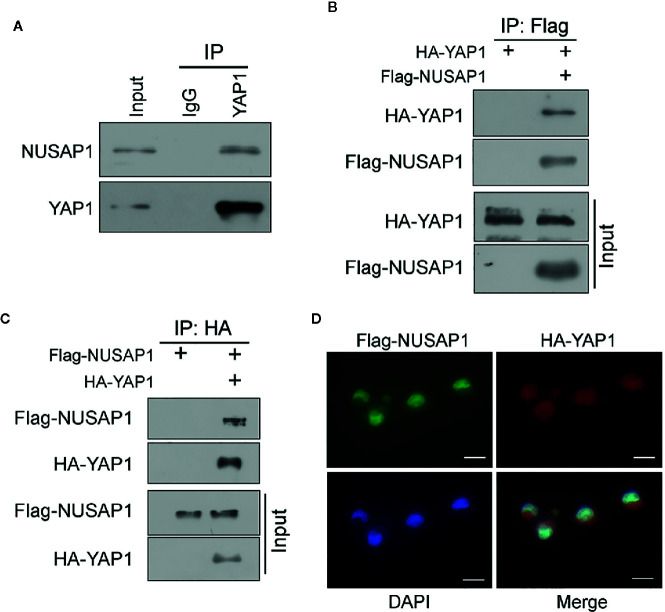
NUSAP1 interacts with YAP1. **(A)** The interaction between endogenous NUSAP1 and YAP1. The BGC823 cell lysates were immunoprecipitated with anti-YAP1 or control immunoglobulin G (IgG), followed by WB analysis with anti-NUSAP1 and anti-YAP1. **(B)** WB analysis of coprecipitating proteins in IPs performed using anti-Flag beads on lysates prepared from BGC823 cells. **(C)** WB analysis of coprecipitating proteins in IPs performed using anti-HA beads on lysates prepared from SGC7901 cells. **(D)** Immunofluorescence of NUSAP1 and YAP1 staining in BGC823 cells (magnification, ×400).

Then, we performed a series of reciprocal co-IP assays to verify the interaction between NUSAP1 and YAP1. As expected, their interaction was detected endogenously using anti-YAP1 protein (**Figure 1A**). Moreover, ectopic NUSAP1 could pull down ectopic YAP1 and vice versa in GC cells (**Figures 1B, C**), further bolstering the specific binding between NUSAP1 and YAP1. In line with these data, the immunofluorescence analysis showed that NUSAP1 and YAP1 were colocalized in the nucleus in GC cells (**Figure 1D**). Taken together, these data demonstrate that NUSAP1 interacts with YAP1 in GC cells.

### NUSAP1 Is Upregulated in GC and Predicts Disease Progression and Poor Prognosis

**Figure 2:**
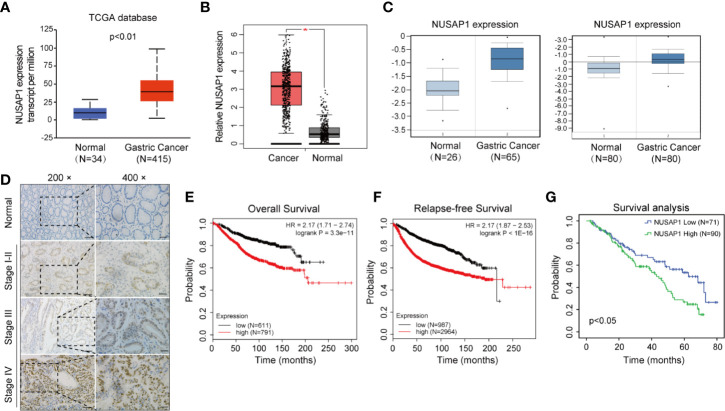
NUSAP1 is upregulated in GC and predicts disease progression and poor prognosis. **(A, B)** Analysis of the relative mRNA expression of NUSAP1 in the TCGA and GEPIA database (Student’s *t* test, *p<0.05). **(C)** The expression profile of NUSAP1 in GC and normal tissues was searched in the Oncomine Gene Browser (Student’s *t* test, *p*<0.05). **(D)** Representative images of IHC staining of NUSAP1 in human GC tissues and non-neoplastic tissue samples. Scale bars represent 50 μM or 25 μM. **(E)** The correlation between NUSAP1 expression and overall survival in patients with GC obtained from Kaplan–Meier Plotter databases (log-rank test, *p*<0.01). **(F)** The correlation between NUSAP1 expression and relapse-free survival (RFS) in patients with GC obtained from Kaplan–Meier Plotter databases (log-rank test, *p*<0.01). **(G)** Kaplan–Meier survival analysis performed with survival data of gastric patients with high NUSAP1 expression (*n*=90) versus low NUSAP1 expression (*n*=71), Log-rank test, *p*=0.012.

We first performed bioinformatic analysis of the online database and found that NUSAP1 was frequently amplified in most types of malignancies, including GC (**Supplementary Figure S2**). Then, we analyzed the expression of NUSAP1 in normal gastric tissues and GC tissues using data deposited in TCGA and the GEPIA database (Gene Expression Profiling Interactive Analysis, http://gepia.cancer-pku.cn/). As shown in **Figures 2A, B**, NUSAP1 was found to be enriched in GC tissues compared to normal gastric tissues. Moreover, analysis of NUSAP1 expression from data deposited from the Oncomine database also revealed that the mRNA expression of NUSAP1 was upregulated in gastric cancer tissues (**Figure 2C**). To further verify the clinical significance and prognostic value of NUSAP1 in GC, we further confirm the association between NUSAP1 expression and the clinicopathological characteristics of GC patients using immunohistochemistry in 161 paraffin-embedded GC tissues. The IHC results reveal that NUSAP1 was upregulated in GC tissues (90/161, 56%), and the staining of NUSAP1 increased markedly with clinical TNM stage (**Figure 2D**), indicating that overexpression of NUSAP1 contributes to GC tumorigenesis and progression. Furthermore, the statistical analysis suggests that its high expression correlates with advanced clinical TNM stage (*p*=0.006), depth of tumor invasion (*p*=0.021), and lymph node metastasis (*p*=0.039) (illustrated clearly in **Table 1**). We also performed survival analysis using data obtained from Kaplan–Meier Plotter databases (http://www.kmplot.com) and found that GC patients with higher NUSAP1 mRNA expression had worse overall survival and relapse-free survival than patients with lower NUSAP1 expression (**Figures 2E, F**; Affymetrix ID: 218039_at). In addition, the clinical follow-up data for these GC patients showed that patients with higher NUSAP1 expression had poorer clinical outcomes (median overall survival, 62.6 months vs. 43 months; *p*<0.05, **Figure 2G**). Thus, our data indicate that NUSAP1 is upregulated in GC and predicts disease progression and poor prognosis.

**Table 1:** The correlation between the expression of NUSAP1 and clinicopathological parameters of patients with GC.

Factors	Cases	NUSAP1 expression		
	(n)	High	Low	P value
Age (years)				
<65	84	44	40	0.347
≥65	77	46	31	
Gender				
Male	97	53	44	0.691
Female	64	37	27	
Tumor Size (cm)				
<5	68	33	35	0.107
≥5	93	57	36	
Differentiation				
Well or Moderately	80	40	40	0.134
poor	81	50	31	
TNM stage^a^				
I-II	69	30	39	0.006
III-IV	92	60	32	
Depth of invasion^a^				
T_1_-T_2_	81	38	43	0.021
T_3_-T_4_	80	52	28	
Tumor location				
Proximal	73	37	36	0.225
Distal	88	53	35	
Lauren classification				
Intestinal type	99	58	41	0.386
Diffuse type	62	32	30	
Lymph node metastasis				
N_0_	76	36	40	0.039
N_X_	85	54	31	

P-values determined using χ^2^ test.

### NUSAP1 Is Required for the Proliferation, Migration, and Invasion of GC Cells *in Vitro*

**Figure 3:**
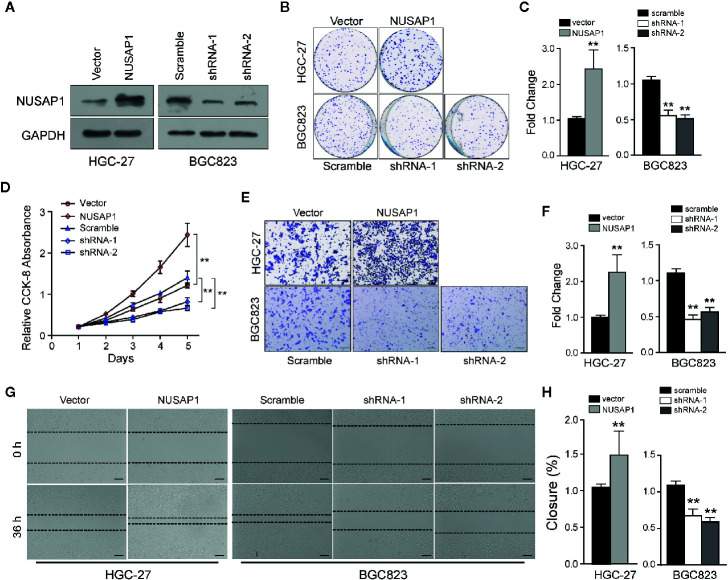
NUSAP1 is required for the proliferation, migration, and invasion of GC cells *in vitro*. **(A)** HGC-27 and BGC823 cells were transfected with Flag-NUSAP1 or NUSAP1 shRNAs, and the efficiency was detected by Western blotting. **(B, C)** Representative images of colony-formation assays for modified HGC-27 and BGC823 cells. Cells were fixed and stained, the colonies were counted, and the data are represented in the bar graph. **(D)** Cell viability was analyzed by CCK-8 assay. **(E, F)** Representative images of fixed and stained modified HGC-27 and BGC823 cells in the Transwell invasion assays (magnification, ×200). **(G, H)** Cell migration ability evaluated by wound-healing assays (magnification, ×100). Student’s *t* test: **p<0.01.

Then, we performed a set of cellular functional experiments to assess the role of NUSAP1 in GC cells. We first confirmed the efficiency of our shRNAs and NUSAP1 expression constructs (**Figure 3A** and **Supplementary Figure S3A**). Growth curves obtained from the CCK-8 assay revealed that NUSAP1 overexpression augmented cell growth in HGC-27 and SGC7901 cells, whereas NUSAP1 knockdown markedly decreased cell growth in BGC823 cells and MGC803 cells (**Figure 3D** and **Supplementary Figure S3D**). These findings were further confirmed by colony-forming assays (**Figures 3B, C** and **Supplementary Figure S3B, C**). Interestingly, NUSAP1 knockdown also markedly decreased the migration and invasion ability of GC cells, and NUSAP1 overexpression enhanced migration and invasion compared to that in control cells (**Figures 3E–H** and **Supplementary Figures S3E–H**). Together, our data indicate that NUSAP1 promotes cell proliferation, migration, and invasion of GC cells *in vitro*.

### NUSAP1 Depletion Impedes Xenograft Tumor Growth *in Vivo*

**Figure 4:**
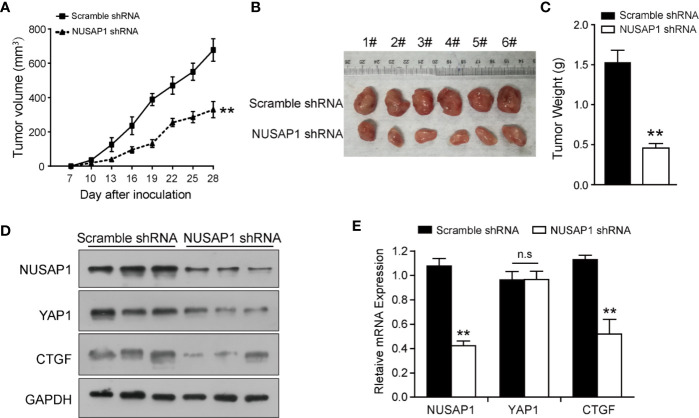
NUSAP1 depletion impedes xenograft tumor growth *in vivo*. **(A)** Growth curves of xenograft tumors derived from BGC823 cells that expressed scramble or NUSAP1 shRNAs. Data are represented as the mean ± SEM, *n* = 6. **(B)** The images of xenograft tumors that were harvested at the end of the experiment. **(C)** Quantification of the average weights of collected tumors from the above experiments. **(D)** The protein levels of NUSAP1, YAP1, and CTGF were detected in six tumors by Western blotting. **(E)** The mRNA levels of NUSAP1, YAP1, and CTGF were detected in six tumors by qRT-PCR (mean ± SEM, *n* = 6). **p<0.01 by two-tailed *t* test.

In order to further confirm the biological functions of NUSAP1, we established a xenograft tumor model by inoculating BGC823 cells that expressed scramble shRNA or NUSAP1 shRNA into NOD/SCID mice and monitored tumor size for 28 days. As illustrated in **Figure 4A**, NUSAP1 knockdown significantly slowed the growth of xenograft tumors. In line with the tumor growth curve, NUSAP1 knockdown resulted in a marked reduction in tumor mass and weight (**Figures 4B, C**). To confirm our cell-based data, we performed Western blotting and qRT-PCR assays using the xenograft tumors. As expected, the protein levels of YAP1 and CTGF were significantly decreased in NUSAP1-shRNA groups (**Figure 4D**). Consistently, the mRNA levels of NUSAP1 and CTGF were significantly decreased upon NUSAP1 knockdown without affecting YAP1 mRNA expression (**Figure 4E**). Collectively, our data indicated that NUSAP1 depletion retarded tumor cell growth *in vivo*.

### NUSAP1 Orchestrates the Hippo Pathway by Stabilizing YAP1 Protein

**Figure 5:**
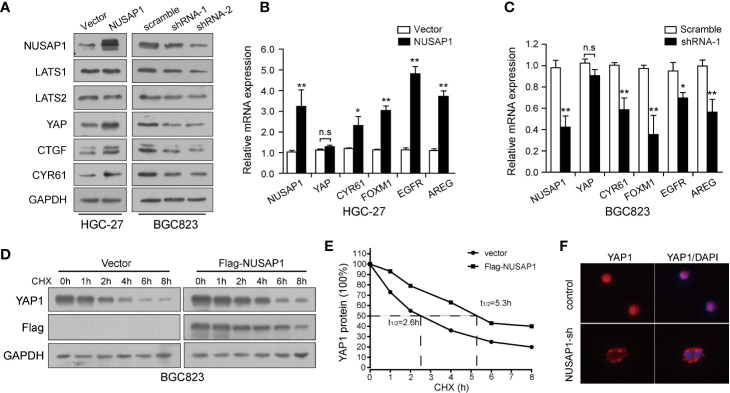
NUSAP1 orchestrates the Hippo pathway by stabilizing YAP1 protein. **(A)** Western blotting analysis to evaluate LATS1/2, YAP1, and YAP1 downstream target genes CTGF and CYR61 in lysates prepared from modified cell lines HGC-27 and BGC823. GAPDH was used as a loading control. **(B, C)** qRT-PCR analysis of YAP1 and its downstream target genes CYR61, FOXM1, EGFR, and AREG in modified HGC-27 and BGC823 cells. Relative expression is shown over GAPDH mRNA. **(D)** Western blotting analysis of YAP1 protein in modified BGC823 cells treated with CHX (25 μg/mL) for the indicated times. **(E)** The line graph shows YAP1 levels normalized to GAPDH at the indicated time points. **(F)** Immunofluorescence of YAP1 in modified BGC823 cells showing cellular localization. Student’s *t* test: *p<0.05, **p<0.01.

Previously, our study demonstrated that the Hippo-YAP1 pathway plays a crucial role in GC tumorigenesis and progression (34, 35); we, therefore, wonder if the interaction between NUSAP1 and YAP1 confers any role to NUSAP1 in regulation of the Hippo–YAP1 pathway. Western blotting analysis of lysates prepared from NUSAP1-modified GC cells showed that the protein expression of YAP1 and target genes, CTGF and CYR61, were closely related to NUSAP1 protein levels. The protein expression of YAP1 and its downstream target genes was reduced in NUSAP1-shRNA cells but increased in HGC-27-NUSAP1 cells (**Figure 5A**) without significant effects on LATS1/2 protein expression. To investigate the mechanisms by which NUSAP1 modulates YAP1 activity, we first examined if NUSAP1 regulates YAP1 transcriptionally. Based on the qRT-PCR results, the mRNA expression of CYR61, FOXM1, EGFR, and AREG correlated with overexpression or knockdown of NUSAP1 in GC cells (**Figures 5B, C**) without affecting YAP1 mRNA expression levels. Because NUSAP1 knockdown affected only the protein but not the mRNA expression of YAP1, we next sought to explore the underlying mechanism using a cycloheximide-chase experiment. As clearly shown in **Figures 5D, E**, ectopic expression of NUSAP1 markedly prolonged YAP1’s half-life from 2.3 to 5.6 h, indicating that NUSAP1 may contribute to YAP1 protein stability in GC cells. Notably, we further found that knockdown of NUSAP1 reduced YAP1 nuclear localization (**Figure 5F** and **Supplementary Figure S4**), leading to YAP1 release to cytoplasm, resulting in its inactivation and degradation. Collectively, our data indicate that NUSAP1 orchestrates the Hippo pathway by stabilizing YAP1 protein.

### YAP1 Functions as a Downstream Mediator of NUSAP1 to Accelerate GC Cell Proliferation and Invasion

**Figure 6:**
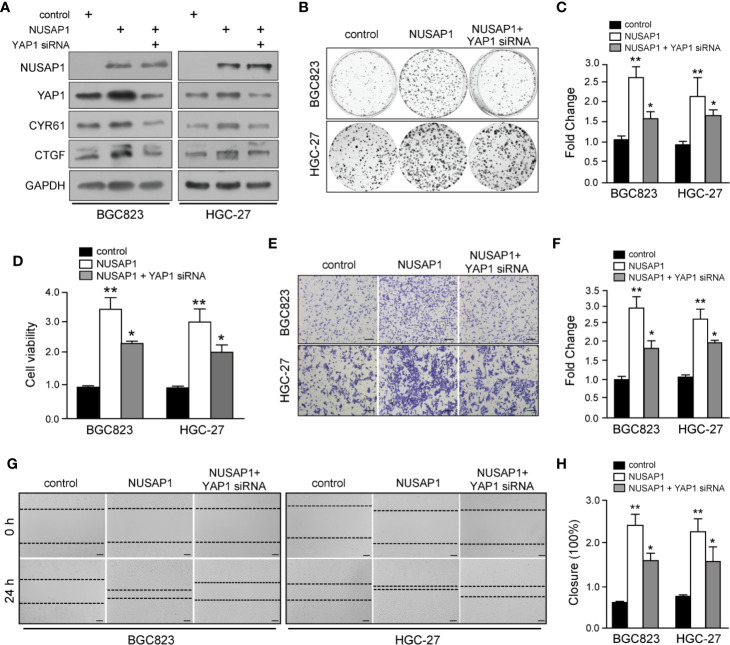
YAP1 functions as a downstream mediator of NUSAP1 to accelerate GC cell proliferation and invasion. **(A)** Western blotting analysis of lysates prepared from NUSAP1-overexpressing GC cells with YAP1 siRNA. GAPDH was used as a loading control. **(B)** Colony formation assay performed on NUSAP-overexpressing BGC823 and HGC-27 cells with YAP1 knockdown. **(C)** Graphic representation of the colony numbers under the indicated transfection condition. **(D)** CCK-8 assay performed on NUSAP-overexpressing BGC823 and HGC-27 cells with YAP1 knockdown. **(E)** Representative images of fixed and stained Transwell invasion assays performed on modified BGC823 and HGC-27 cells (magnification, ×200). **(F)** Graphic representation of invaded cell counts from the Transwell assays. **(G, H)** Cell migration ability evaluated by wound-healing assays performed on modified BGC823 and HGC-27 cells (magnification, ×200). One-way analysis of variance (adjusted for Bonferroni correction test). All data are presented as the mean ± SEM of triplicate experiments, *n*=3, *P<0.05 and **P<0.01.

To further determine the role of YAP1 in NUSAP1-mediated oncogenesis in GC, we knocked down YAP1 using siRNA with or without NUSAP1 overexpression in BGC823 and HGC-27 cells. As shown in **Figure 6A**, knockdown of YAP1 reduced CTGF and CYR61 protein expression without significant effect on NUSAP1 expression in GC cells. Strikingly, YAP1 deficiency partially negated the effects of NUSAP1 on the protein levels of YAP1 as well as its downstream targets CTGF and CYR61 (**Figure 6A**). Furthermore, CCK-8, colony-formation, wound-healing, and transwell invasion assays further indicated that NUSAP1-induced proliferation, migration, and invasion were partially reversed by YAP1 depletion in BGC823 and HGC-27 cells (**Figures 6B–H**). Taken together, these findings demonstrated that YAP1 acts as a main downstream factor of NUSAP1 to promote the proliferation and invasion ability of GC cells.

### NUSAP1 Expression Positively Correlates With YAP1 Protein Levels in GC

**Figure 7:**
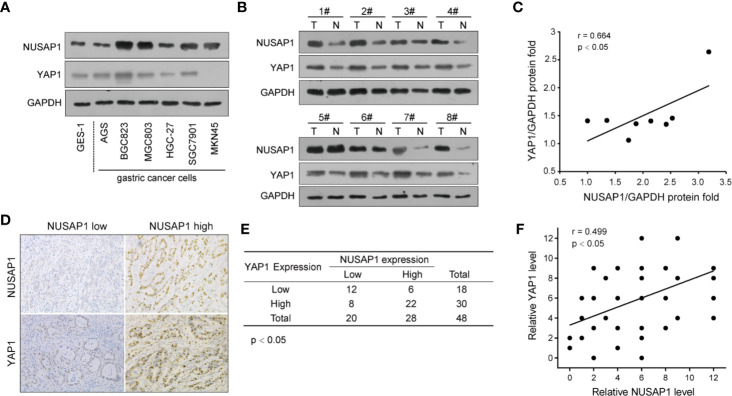
NUSAP1 expression positively correlates with YAP1 protein levels in GC. **(A)** Western blotting analysis of NUSAP1 and YAP1 levels in GES-1, AGS, BGC823, MGC803, HGC-27, SGC7901, and MKN45 cell lines. GAPDH was used as a loading control. **(B)** Western blotting analysis of NUSAP1 levels in lysates prepared from 8 paired GC tissues and normal gastric tissues. **(C)** The correlation analysis of NUSAP1 and YAP1 expression levels in 8 GC patients. **(D)** Representative immunochemical staining of NUSAP1 and YAP1 in GC tissues. **(E, F)** Correlation of NUSAP1 expression with YAP1 expression in primary 48 GC patients (Pearson’s correlation coefficient, the chi-square test, *p*<0.01).

To further verify the relevance of YAP1 regulation by NUSAP1 in GC, we conducted the Western blotting analysis of NUSAP1 and YAP1 expression in GC cell lines and tissues. As illustrated in **Figure 7A**, high NUSAP1 protein levels associated with increased YAP1 expression in GC cell lines when compared with GES-1 cells. Moreover, we also detect their expression in 8 pairs of clinical GC tissues and their adjacent normal tissues (**Figure 7B**). The expression of NUSAP1was relatively higher in GC tissues, which showed much more increased YAP1 expression. The statistical analysis revealed a positive correlation between NUSAP1 and YAP1 protein expression (**Figure 7C**; *r*=0.664, *p*<0.05), suggesting that NUSAP1 overexpression is positively correlated with YAP1 levels. We also undertook immunohistochemistry assays to evaluate the protein expression of NUSAP1 and YAP1 in 48 GC patients. As shown in **Figure 7D**, high NUSAP1 levels correlated with increased YAP1 expression in most GC specimens. Notably, 34 (70%) tissue specimens showed simultaneously high or low expression of NUSAP1 and YAP1 (**Figures 7E, F**; *p*<0.05, *r*=0.499). These data reveal that NUSAP1 expression positively correlates with YAP1 protein in GC cell lines and tissues.

## Discussion

In this study, we newly determine that NUSAP1 functions as a positive regulator of YAP1 to facilitate GC tumorigenesis and progression. Our data show that NUSAP1 is markedly upregulated in GC, and elevated NUSAP1 is an indicator for disease progression and poor survival in patients with GC. Specifically, NUSAP1 exerts cancer-promoting functions in GC cells, and these effects are partially reversed by YAP1 depletion. With regard to the molecular mechanism, we discovered for the first time that NUSAP1 enhances YAP1 protein stability by physically interacting with YAP1. This study provides a deeper understanding of the intricate regulatory network of the YAP1 oncogene, and this clarifies that the NUSAP1–YAP1 signaling axis is critical in the progression and tumorigenesis of GC.

There is compelling evidence demonstrating that the Hippo–YAP1 pathway ranks as the most prominent pathway in gastric oncogenesis (36). Other and our previous works delineate the role and the regulation mechanism of YAP1 in GC (37, 38). In this study, we, for the first time, identify NUSAP1 as a new YAP1-binding protein using Co-IP and immunofluorescence assays. NUSAP1 upregulation is detected in a broad range of human cancers, such as prostate cancer, astrocytoma, lung cancer, and breast cancer (20, 21, 23, 25). For instance, NUSAP1 is highly overexpressed in colon and prostate cancers and closely correlated with poor prognosis (39, 40). Results from others have further revealed that patients with high NUSAP1 expression levels were strongly related to unfavorable clinical characteristics and promoted cell proliferation and invasion in GC (26). Here, we consistently report that NUSAP1 is upregulated in 56% of GC cases and correlates with TNM stage, depth of invasion, and lymph node metastasis, potentially leading to the onset of GC. Different from other reports, we have not identified the correlation between NUSAP1 expression and tumor size, but our study first proposes that NUSAP1 functions as a prognostic factor for overall survival in patients with GC. Moreover, we demonstrate that overexpression of NUSAP1 strongly accelerates GC cell proliferation, migration, and invasion, which are suppressed by NUSAP1 depletion. These data suggest that NUSAP1 functions as a potent oncogenic player in human GC. However, the potential mechanism by which NUSAP1 exerts oncogenic roles remains unclear.

In the present study, we find that NUSAP1 has a profound effect on the Hippo–YAP1 pathway. NUSAP1 overexpression upregulates the expression of downstream targets of the Hippo pathway, including YAP1, CYR61, CTGF, and FOXM1, in GC cells. In contrast, knockdown of NUSAP1 markedly decreases the protein expression of YAP1 and the downstream target genes. The YAP1 protein expression level is canonically regulated by the LATS1/2 kinase of the Hippo pathway and is critical for tumor initiation, progression, and metastasis (32, 41). The fact that LATS1/2 protein did not change markedly in response to modulation of NUSAP1 expression indicates that the regulation of YAP1 by NUSAP1 is independent of LATS kinase. More interestingly, neither overexpression nor depletion of NUSAP1 caused significant effects on YAP1 mRNA levels, suggesting that NUSAP1 regulates YAP1 function through post-transcriptional or protein degradation levels in GC cells. Stabilization of YAP1 protein expression is one of the mechanisms by which YAP1 exerts its various functions (42, 43). Here, we demonstrate that NUSAP1 prolongs YAP1’s protein half-life *via* interaction with YAP1, resulting in activation of the transcription of Hippo pathway downstream target genes. This NUSAP1-YAP1 complex may recruit YAP1 from the cytoplasm to the nucleus as NUSAP1 depletion released YAP1 to cytoplasm. However, how NUSAP1 directly or indirectly affects the translocation of YAP1 remains unanswered. Previous studies show that the recruitment of β-TrCP ubiquitin ligase to the C-terminal region of YAP1 facilitates its ubiquitination and degradation (44). We suspect that NUSAP1 interacts with the C-terminal region of YAP1, preventing it from being ubiquitinated by β-TrCP ligase, thus leading to YAP1 stabilization and activation. More studies to delineate these questions are of great interest in the future.

More convincingly, the effects of NUSAP1 on GC progression could be partially reversed by YAP1 depletion, suggesting that YAP1 acts as a main downstream factor of NUSAP1. However, our data reveal that NUSAP1-mediated cell proliferation and invasion could not be wholly rescued by YAP1 depletion, suggesting that YAP1 is not the sole downstream mediator of NUSAP1 in GC cells. NUSAP1 has been demonstrated to regulate several cancer-promoting genes and pathways, including BRCA1, Wnt/β-catenin, mTORC1, Hedgehog, etc (22, 25, 26, 45). These lines of evidence indicate that NUSAP1 may manipulate these various oncogenes and pathways in GC. Of note, a significant positive correlation between the NUSAP1 and YAP1 protein levels in GC tissues was validated in our study, further suggesting that overexpression of NUSAP1 might contribute to YAP1 activation in GC. However, it should be noticed that a small fraction of human gastric tumors had low NUSAP1 expression but high YAP1 expression, indicating that YAP1 can be activated by other mechanisms, including phosphorylation or ubiquitination, such as NLK or YOD1 (46, 47).

In summary, our study unveils a new connection between NUSAP1 and the Hippo–YAP1 signaling pathway. NUSAP1 binds and stabilizes YAP1, thereby orchestrating the Hippo pathway, leading to GC tumorigenesis and progression.

## Data Availability Statement

The raw data supporting the conclusions of this article will be made available by the authors, without undue reservation.

## Ethics Statement

The studies involving human participants were reviewed and approved by the Independent Ethics Committee of the First Affiliated Hospital of Nanchang University. The patients/participants provided their written informed consent to participate in this study. The animal study was reviewed and approved by the Independent Ethics Committee of the First Affiliated Hospital of Nanchang University.

## Author Contributions

HG and JZ conducted the laboratory experiments and acquisition of data and technical support. LZ and YH contributed to analysis and technical support. MZ and JL contributed to collection and analysis of clinical samples. SH and JC revised the manuscript for important intellectual content. JX and ZF contributed to study concept and design, analysis, and interpretation of data and drafting of the manuscript. XX contributed to analysis and interpretation of data and drafting of the manuscript. All authors contributed to the article and approved the submitted version.

## Funding

Our study was funded by the National Natural Science Foundation of China (grants 81660402, 81860545 and 82060566), the Natural Science Foundation of JiangXi Province (grants 20192BAB215040, 20192BAB205073 and 20202ACBL216011), the Jiangxi Provincial Outstanding Young Talents projects (grant 20192BCB23020) and Education Department of Jiangxi Province (grant 701238001) and Department of Health Project of Jiangxi Province (grant 20201015). This manuscript has been released as a preprint at ResearchSquare, https://www.researchsquare.com/article/rs-33883/v1 (48).

## Conflict of Interest

The authors declare that the research was conducted in the absence of any commercial or financial relationships that could be construed as a potential conflict of interest.

## Supplementary Material

Supplementary Figure 1 List of several YAP1-associated proteins identified by mass spectrometric analysis.

Supplementary Figure 2 Analysis of the database in GeneMANIA shows that NUSAP1 was amplified in multiple malignancies.

Supplementary Figure 3 NUSAP1 regulates cell proliferation, migration, and invasion of GC cells *in vitro*. **(A)** SGC7901 and MGC803 cells were transfected with Flag-NUSAP1 or NUSAP1 shRNAs, and the transfection efficiency was detected by Western blotting. **(B, C)** Representative images of colony-formation assays for modified SGC7901 and MGC803 cells. Cells were fixed and stained, the colonies were counted, and the data are represented in the bar graph. **(D)** Cell viability was analyzed by CCK-8 assay. **(E, F)** Representative images of fixed and stained modified SGC7901 and MGC803 cells in the Transwell invasion assays (magnification, ×200). **(G, H)** Cell migration ability evaluated by wound-healing assays (magnification, ×100). Student’s *t* test: **p*<0.05, ***p*<0.01.

Supplementary Figure 4 Immunofluorescence of YAP1 in modified SGC7901 cells showing cellular localization.
