# Corrigendum: NUSAP1 Promotes Gastric Cancer Tumorigenesis and Progression by Stabilizing the YAP1 Protein

**DOI:** 10.3389/fonc.2021.666560

**Published:** 2021-03-23

**Authors:** Hui Guo, Jianping Zou, Ling Zhou, Min Zhong, Yan He, Shanshan Huang, Jun Chen, Junhe Li, Jianping Xiong, Ziling Fang, Xiaojun Xiang

**Affiliations:** Department of Oncology, The First Affiliated Hospital of Nanchang University, Nanchang, China

**Keywords:** gastric cancer, NUSAP1, YAP1, protein stability, tumorigenesis and progression

In the original article, there was a mistake in Figure 1 as published. Since the blots initially used for Figure 1C could not be located in our records, as they were generated by Hui Guo in 2016, who has graduated and left our lab, we decided to use its repeated experimental result done by Jianping Zou as a replacement. The corrected Figure 1 appears below.

**Figure 1:**
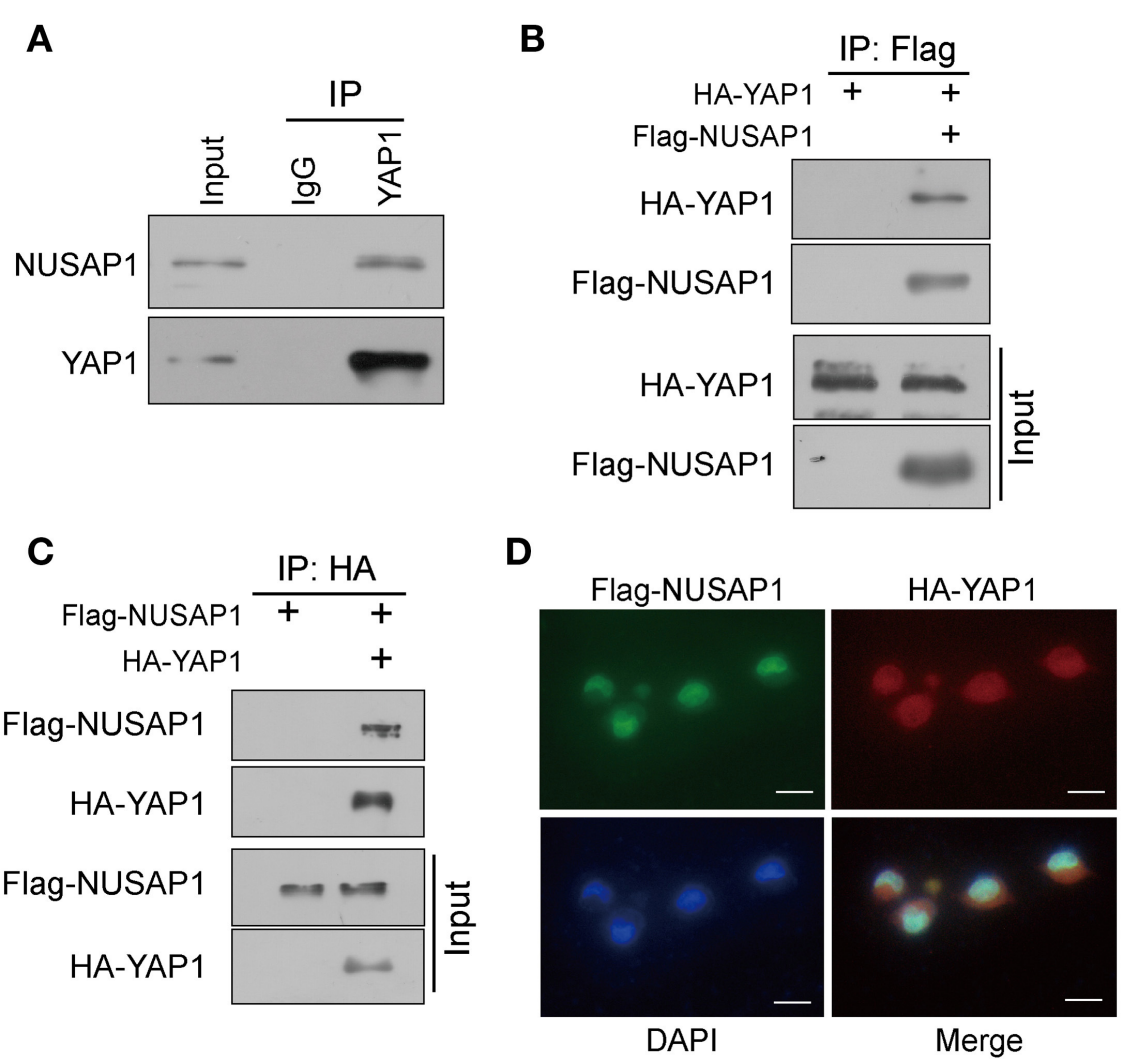
NUSAP1 interacts with YAP1. **(A)** The interaction between endogenous NUSAP1 and YAP1. The BGC823 cell lysates were immunoprecipitated with anti-YAP1 or control immunoglobulin G (IgG), followed by WB analysis with anti-NUSAP1 and anti-YAP1. **(B)** WB analysis of coprecipitating proteins in IPs performed using anti-Flag beads on lysates prepared from BGC823 cells. **(C)** WB analysis of coprecipitating proteins in IPs performed using anti-HA beads on lysates prepared from SGC7901 cells. **(D)** Immunofluorescence of NUSAP1 and YAP1 staining in BGC823 cells (magnification, ×400).

There was also a mistake in Figure 3 as published. This was due to a mistake when handling transwell images in Adobe Illustrator. The corrected Figure 3 appears below.

**Figure 3:**
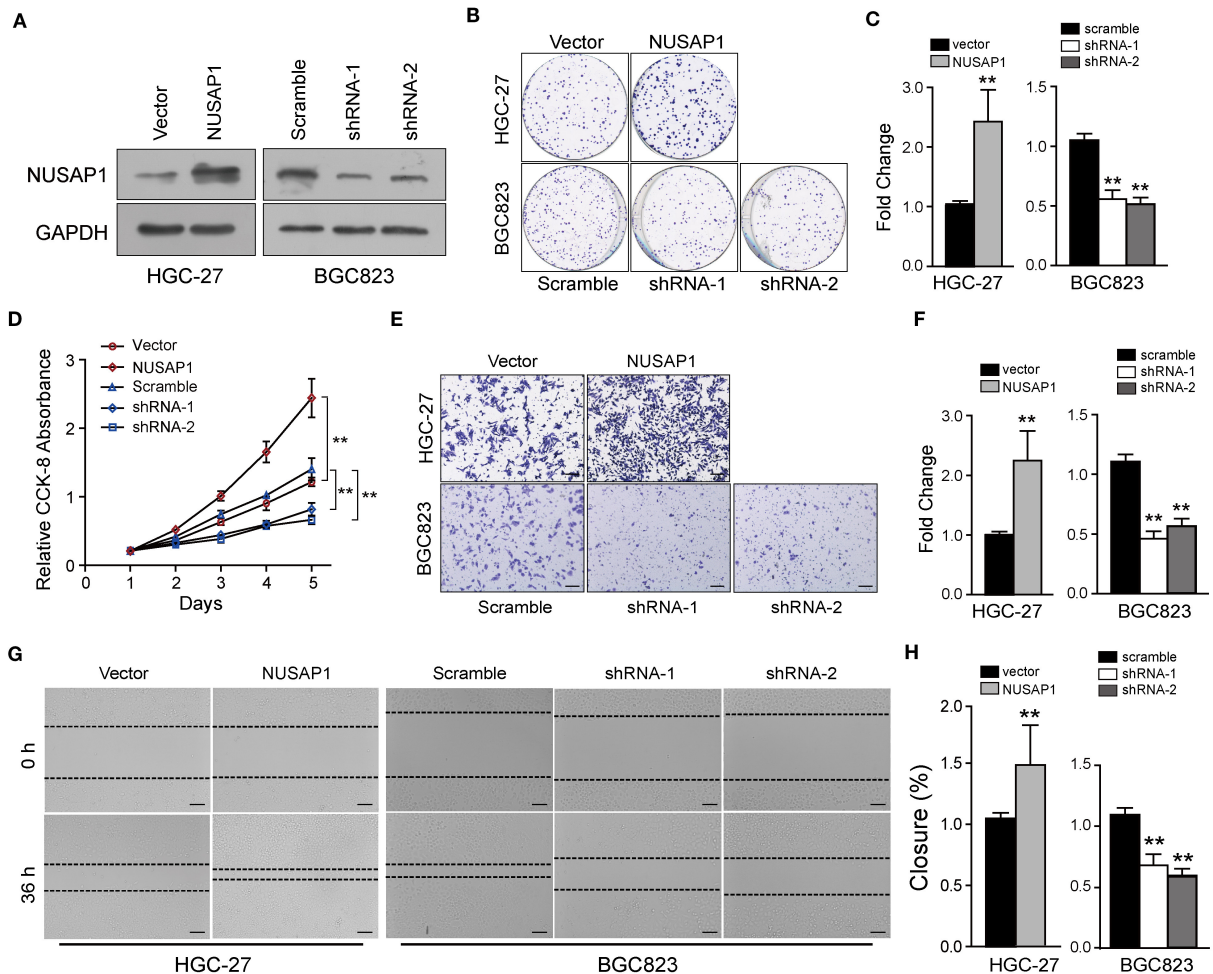
NUSAP1 is required for the proliferation, migration, and invasion of GC cells *in vitro*. **(A)** HGC-27 and BGC823 cells were transfected with Flag-NUSAP1 or NUSAP1 shRNAs, and the efficiency was detected by Western blotting. **(B, C)** Representative images of colony-formation assays for modified HGC-27 and BGC823 cells. Cells were fixed and stained, the colonies were counted, and the data are represented in the bar graph. **(D)** Cell viability was analyzed by CCK-8 assay. **(E, F)** Representative images of fixed and stained modified HGC-27 and BGC823 cells in the Transwell invasion assays (magnification, ×200). **(G, H)** Cell migration ability evaluated by wound-healing assays (magnification, ×100). Student's *t* test: **p < 0.01.

The authors apologize for these errors and state that these do not change the scientific conclusions of the article in any way. The original article has been updated.

