# Supplementary figures and images for: NUSAP1 Promotes Gastric Cancer Tumorigenesis and Progression by Stabilizing the YAP1 Protein
Source: Front Oncol. 2021 Jan 7;10:591698. doi: 10.3389/fonc.2020.591698 (PMC7817543; doi:10.3389/fonc.2020.591698)

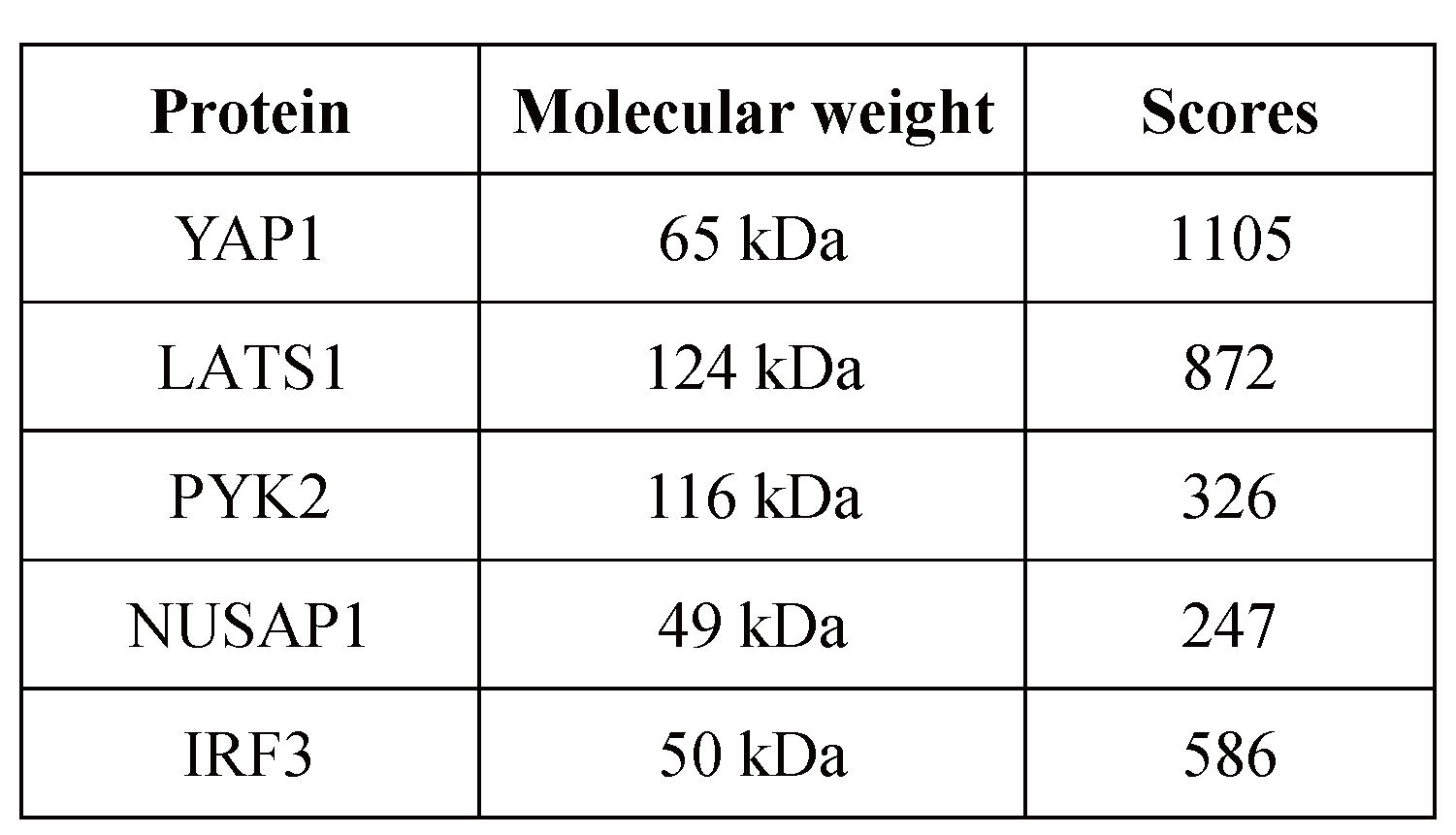

Supplement: Supplementary Figure 1 — List of several YAP1-associated proteins identified by mass spectrometric analysis. [file Image_1.jpeg]

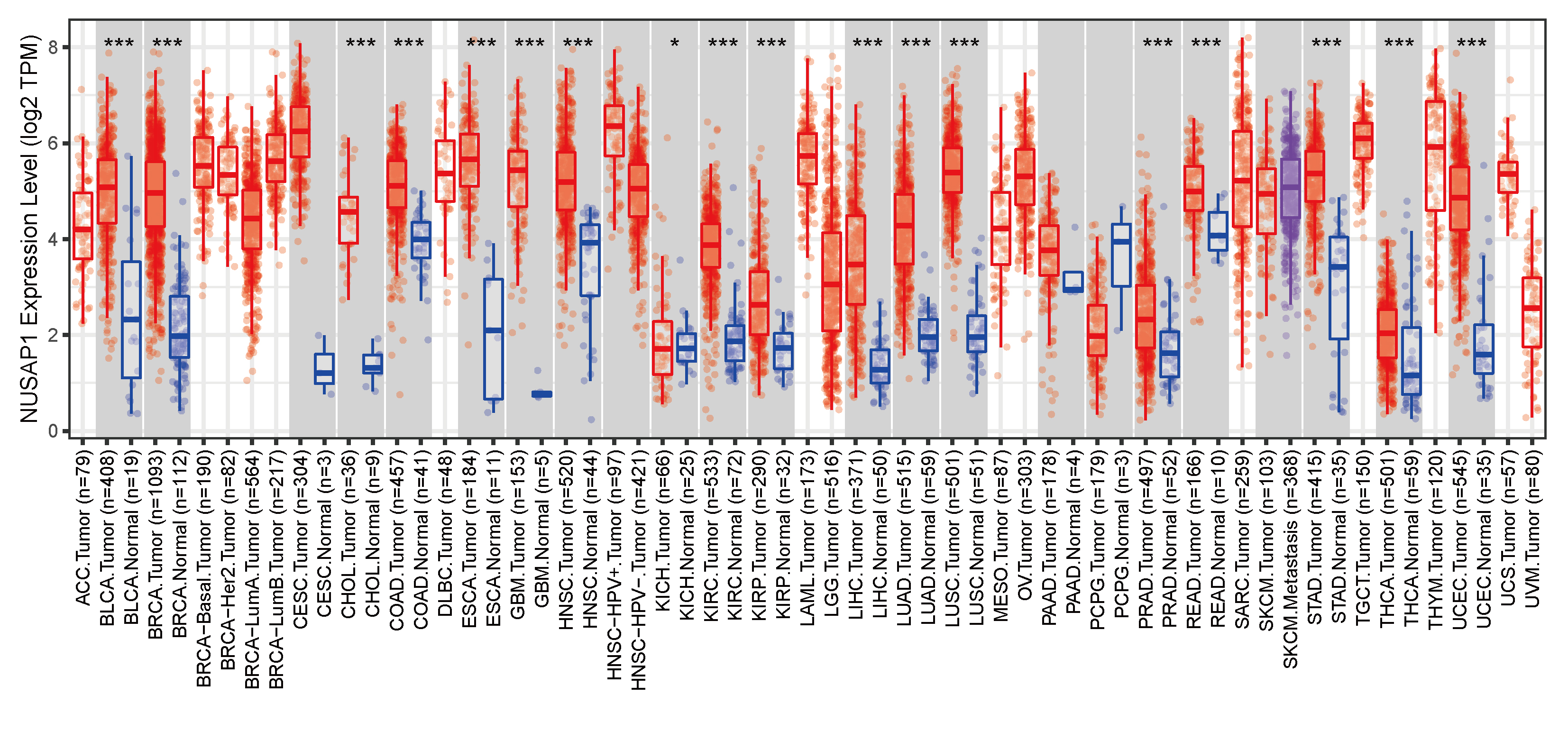

Supplement: Supplementary Figure 2 — Analysis of the database in GeneMANIA shows that NUSAP1 was amplified in multiple malignancies. [file Image_2.jpeg]

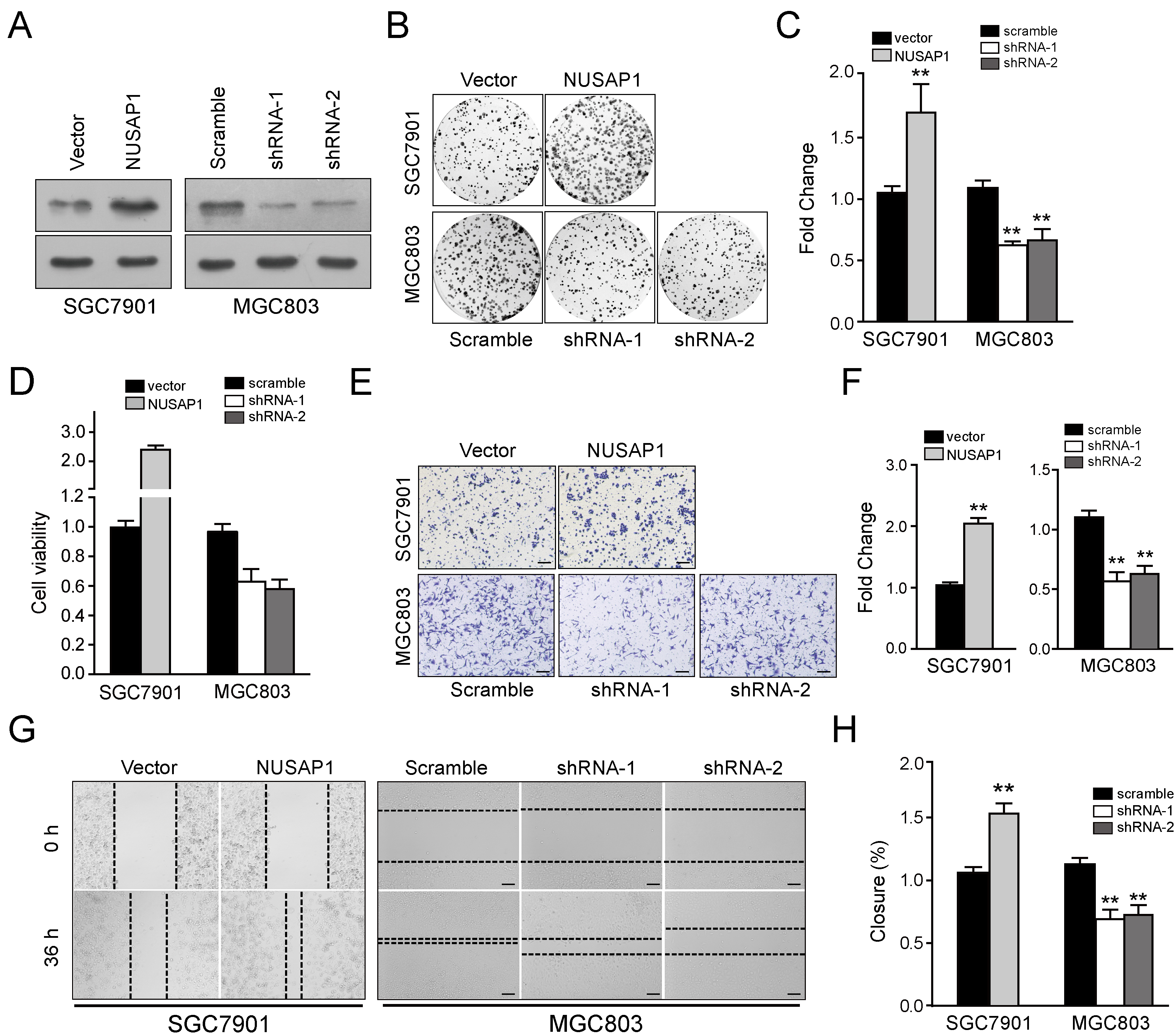

Supplement: Supplementary Figure 3 — NUSAP1 regulates cell proliferation, migration, and invasion of GC cells in vitro. (A) SGC7901 and MGC803 cells were transfected with Flag-NUSAP1 or NUSAP1 shRNAs, and the transfection efficiency was detected by Western blotting. (B, C) Representative images of colony-formation assays for modified SGC7901 and MGC803 cells. Cells were fixed and stained, the colonies were counted, and the data are represented in the bar graph. (D) Cell viability was analyzed by CCK-8 assay. (E, F) Representative images of fixed and stained modified SGC7901 and MGC803 cells in the Transwell invasion assays (magnification, ×200). (G, H) Cell migration ability evaluated by wound-healing assays (magnification, ×100). Student’s t test: *p<0.05, **p<0.01. [file Image_3.jpeg]

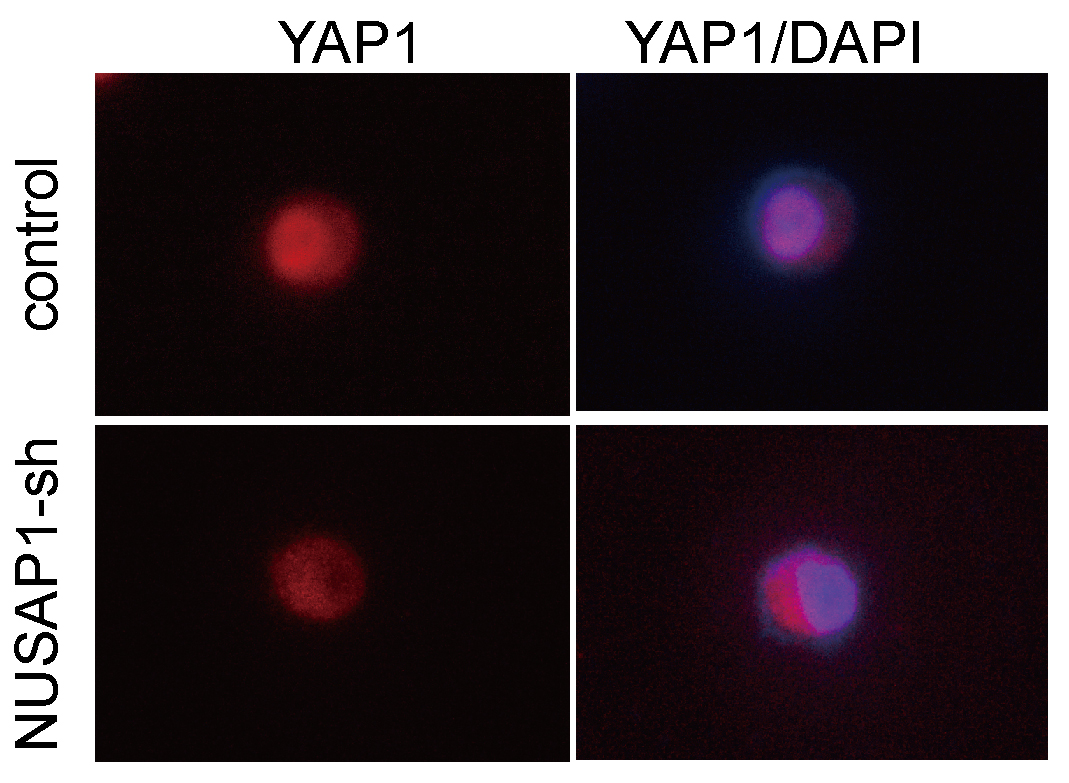

Supplement: Supplementary Figure 4 — Immunofluorescence of YAP1 in modified SGC7901 cells showing cellular localization. [file Image_4.jpeg]
