# Supplementary material for: NUSAP1 Promotes Gastric Cancer Tumorigenesis and Progression by Stabilizing the YAP1 Protein
Source: Front Oncol. 2021 Jan 7;10:591698. doi: 10.3389/fonc.2020.591698 (PMC7817543; doi:10.3389/fonc.2020.591698)
Supplement: Supplementary file 5 [file Table_1.docx]

**Supplementary Table S1: The clinicopathological characteristics of gastric cancer patients.**

| **Patients** | **Age (year)** | **gender** | **Tumor size (cm)** | **Depth of tumor invasion** | **Differentiation**  **(well, moderate and poorly)** | **TNM stage** | **Lymph node metastasis**  **(N_0_ or N_X_)** |
| --- | --- | --- | --- | --- | --- | --- | --- |
| #1 | 64 | Male | 2.8*3.3 | T_3_ | moderate | II | N_0_ |
| #2 | 54 | Male | 3.2*2.5 | T_2_ | poorly | III | N_X_ |
| #3 | 53 | Female | 2.9*1.5 | T_2_ | moderate | I | N_0_ |
| #4 | 66 | Male | 2.3*4.2 | T_4_ | poorly | III | N_0_ |
| #5 | 53 | Female | 1.8*2.0 | T_1_ | well | I | N_0_ |
| #6 | 62 | Female | 2.4*1.8 | T_2_ | moderate | II | N_0_ |
| #7 | 50 | Male | 4.7*5.3 | T_4_ | Poorly | IV | N_X_ |
| #8 | 55 | Female | 4.3*2.7 | T_4_ | poorly | III | N_X_ |
