# Supplementary material for: NUSAP1 Promotes Gastric Cancer Tumorigenesis and Progression by Stabilizing the YAP1 Protein
Source: Front Oncol. 2021 Jan 7;10:591698. doi: 10.3389/fonc.2020.591698 (PMC7817543; doi:10.3389/fonc.2020.591698)
Supplement: Supplementary file 6 [file Table_2.docx]

**Supplementary Table S2: The sequences of NUSAP1 shRNAs and YAP siRNAs used in this study.**

| **Gene** | **Sequences for shRNAs** |
| --- | --- |
| NUSAP1 shRNA-1 | 5′-GCACCAAGAAGCTGAGAATGC-3′ |
| NUSAP1 shRNA-2 | 5′-GGAAATGGAGTCCATTGATCA-3′ |
| YAP siRNA | 5′-CUGCCACCAAGCUAGAUAATT-3′ |
