# Supplementary material for: NUSAP1 Promotes Gastric Cancer Tumorigenesis and Progression by Stabilizing the YAP1 Protein
Source: Front Oncol. 2021 Jan 7;10:591698. doi: 10.3389/fonc.2020.591698 (PMC7817543; doi:10.3389/fonc.2020.591698)
Supplement: Supplementary file 7 [file Table_3.docx]

**Supplementary Table S3. Primer sequences used in qRT-PCR assays.**

| gene | Primer sequences |
| --- | --- |
| NUSAP1 | Forward: 5’-CTGACCAAGACTCCAGCCAGAA-3’ |
|  | Reverse: 5’-GAGTCTGCGTTGCC TCAGTTGT-3’ |
| YAP | Forward: 5’-TCGTTTTGCCATGAACCAGA-3’ |
|  | Reverse: 5’-GGCTGCTTCACTGGAGCACT-3’ |
| CYR61 | Forward: 5’-CAGGACTGTGAAGATGCGGT-3’ |
|  | Reverse: 5’-GCCTGTAGAAGGGAAACGCT-3’ |
| FOXM1 | Forward: 5’-CACCCATCACCAGCTTGTTT-3’ |
|  | Reverse: 5’-GGAGATTGGGACGAATCCTC-3’ |
| AREG | Forward: 5’-TGTCGCTCTTGATACTCGGC-3’ |
|  | Reverse: 5’-ATGGTTCACGCTTCCCAGAG-3’ |
| EGFR | Forward: 5’-AGGCACGAGTAACAAGCTCAC-3’ |
|  | Reverse: 5’-ATGAGGACATAACCAGCCACC-3’ |
| GAPDH | Forward: 5’-GATTCCACCCATGGCAAATTC-3’ |
|  | Reverse: 5’-AGCATCGCCCCACTTGATT-3’ |
